# Deep Learning-Based Framework for *In Vivo* Identification of Glioblastoma Tumor using Hyperspectral Images of Human Brain

**DOI:** 10.3390/s19040920

**Published:** 2019-02-22

**Authors:** Himar Fabelo, Martin Halicek, Samuel Ortega, Maysam Shahedi, Adam Szolna, Juan F. Piñeiro, Coralia Sosa, Aruma J. O’Shanahan, Sara Bisshopp, Carlos Espino, Mariano Márquez, María Hernández, David Carrera, Jesús Morera, Gustavo M. Callico, Roberto Sarmiento, Baowei Fei

**Affiliations:** 1 Department of Bioengineering, The University of Texas at Dallas, 800 W. Campbell Road, Richardson, TX 75080, USA; martin.halicek@gatech.edu (M.H.); mshahedi@utdallas.edu (M.S.); 2 Institute for Applied Microelectronics (IUMA), University of Las Palmas de Gran Canaria (ULPGC), 35017 Las Palmas de Gran Canaria, Spain; sortega@iuma.ulpgc.es (S.O.); roberto@iuma.ulpgc.es (R.S.); 3 Department of Biomedical Engineering, Emory University and Georgia Institute of Technology, 1841 Clifton Road NE, Atlanta, GA 30329, USA; 4 Department of Neurosurgery, University Hospital Doctor Negrin of Gran Canaria, Barranco de la Ballena s/n, 35010 Las Palmas de Gran Canaria, Spain; adamszolna@wp.pl (A.S.); pinerbrains1@yahoo.es (J.F.P.); coralia.sosa@gmail.com (C.S.); aruosha@gmail.com (A.J.O.); sarabisshop@hotmail.com (S.B.); carlosespinopostigo@gmail.com (C.E.); marquezrdguez@yahoo.es (M.M.); hhdez.maria@gmail.com (M.H.); david__carrera@hotmail.com (D.C.); jmormol@gobiernodecanarias.org (J.M.); 5 Advanced Imaging Research Center, University of Texas Southwestern Medical Center, 5323 Harry Hine Blvd, Dallas, TX 75390, USA; 6 Department of Radiology, University of Texas Southwestern Medical Center, 5323 Harry Hine Blvd, Dallas, TX 75390, USA

**Keywords:** brain tumor, cancer surgery, hyperspectral imaging, bioinformatics, intraoperative imaging, deep learning, precision medicine, image-guided surgery

## Abstract

The main goal of brain cancer surgery is to perform an accurate resection of the tumor, preserving as much normal brain tissue as possible for the patient. The development of a non-contact and label-free method to provide reliable support for tumor resection in real-time during neurosurgical procedures is a current clinical need. Hyperspectral imaging is a non-contact, non-ionizing, and label-free imaging modality that can assist surgeons during this challenging task without using any contrast agent. In this work, we present a deep learning-based framework for processing hyperspectral images of *in vivo* human brain tissue. The proposed framework was evaluated by our human image database, which includes 26 *in vivo* hyperspectral cubes from 16 different patients, among which 258,810 pixels were labeled. The proposed framework is able to generate a thematic map where the parenchymal area of the brain is delineated and the location of the tumor is identified, providing guidance to the operating surgeon for a successful and precise tumor resection. The deep learning pipeline achieves an overall accuracy of 80% for multiclass classification, improving the results obtained with traditional support vector machine (SVM)-based approaches. In addition, an aid visualization system is presented, where the final thematic map can be adjusted by the operating surgeon to find the optimal classification threshold for the current situation during the surgical procedure.

## 1. Introduction

Cancer is a leading cause of mortality worldwide [1]. In particular, brain tumor is one of the most deadly forms of cancer, while high-grade malignant glioma is the most common form (~30%) of all brain and central nervous system tumors [2]. Within these malignant gliomas, glioblastoma (GBM) is the most aggressive and invasive type, accounting for 55% of these cases [3,4]. Current diagnoses of brain tumors are based on excisional biopsy followed by histology. GBM are extremely invasive with potential complications and side effects for the patient [5]. Furthermore, it is not possible to obtain the diagnostic information in real-time during the surgical procedure, since the tissue must be processed in a pathological laboratory.

It has been demonstrated that tumor tissue left behind during surgery of high-grade tumors is the most common cause of tumor recurrence, and is a major cause of morbidity and mortality [5,6,7]. In addition, it has been proven that a complete resection for low-grade tumors is highly beneficial for the outcomes of the patient, especially in pediatric cases [8]. Nowadays, several imaging modalities are used as guidance tools during brain tumor surgeries; nevertheless, these systems have various limitations. Intraoperative neuronavigation systems work with preoperative image data, such as computed tomography (CT) or magnetic resonance imaging (MRI), to guide the surgery in real-time; however, the accuracy of the tumor margin delineation falls as the surgical procedure advances due to the brain shift phenomenon [9,10,11,12]. Intraoperative MRI helps solve the problem of brain shift and is able to monitor the tissue intraoperatively. However, this technique prolongs the surgery duration and requires a specific operating room with MRI-compatible equipment, presenting several challenges to anesthesiologists regarding patient safety, monitoring, and equipment monitoring [13]. Additionally, the refreshing rate of intraoperative MRI is much lower than the refreshing rate of hyperspectral imaging (HSI). Other systems are based on fluorescent tumor markers, such as 5-aminolevulinic acid (5-ALA), providing the detection of the tumor margins intraoperatively. Nonetheless, this technique may produce significant, unwanted side effects for the patient, is not recommended for children, and can only be employed for high-grade tumors [14,15]. In order to solve the limitations found in the current employed systems, hyperspectral imaging (HSI), also known as imaging spectroscopy [16], arises as a non-contact and label-free, non-ionizing, and real-time potential solution that enables accurate detection of the malignant tissue margins, offering a reliable guidance for diagnosis during surgical interventions and treatment [17,18,19,20].

HSI is the term that is designated to the technology that integrates conventional imaging and spectroscopy methods to obtain both the spatial and spectral information of an object [21]. Traditionally, HSI has been widely employed in the remote sensing field [22], and therefore, the majority of algorithms that have been developed to classify HS images are related with this field [23]. However, in the past decades, HSI has been commonly used in other fields, such as drug analysis [24,25], food quality inspection [26,27,28,29] or defense and security [30,31], among many others. In the medical field, this imaging modality has been used for research purposes since the 1990s [32,33,34]. However, in recent years, medical HSI has started to achieve promising results with respect to cancer detection [17,35].

Previous works have studied the classification and delineation of the tumor margins using HSI and traditional machine learning (ML) algorithms [20,36,37,38,39]. Particularly, head and neck cancer were widely investigated using quantitative HSI to identify and outline the tumor boundaries in *in vivo* animal samples employing ML techniques [40,41,42,43], and in *ex vivo* human samples by means of deep learning (DL) techniques [44]. Brain tumors were also investigated, performing quantitative and qualitative HSI analysis with the goal of delineating tumor boundaries and performing an intrapatient validation by employing both the spatial and spectral features of HSI [38]. In addition, qualitative interpatient validation was performed to identify brain tumor areas intraoperatively by using ML algorithms [20].

In this study, a deep learning-based framework has been developed with the goal of creating a surgical aid visualization system that is capable of generating a thematic map where the parenchymal area of the brain is delineated, and the location of the tumor is identified using *in vivo* human brain hyperspectral images. This framework could assist neurosurgeons in the critical task of identifying cancer tissue during brain surgery with higher accuracy than using ML techniques.

## 2. Materials and Methods

This section presents the HSI instrumentation that is employed to obtain the *in vivo* HS brain cancer image database, the deep learning techniques and the proposed pipeline that is developed in this work, the SVM-based approaches that have been used for the comparison of the results, and the validation metrics that have been employed for this comparison.

### 2.1. Intraoperative Hyperspectral Acquisition System

A customized intraoperative hyperspectral (HS) acquisition system [20] was employed to create the HS image database. The system employs a VNIR (visible and near-infrared) pushbroom camera (Hyperspec^®^ VNIR A-Series, Headwall Photonics Inc., Fitchburg, MA, USA) to obtain HS images in the spectral range comprised between 400–1000 nm. This camera generates HS cubes of 826 spectral bands with a FWHM (full width at half maximum) spectral resolution of two to three nm, and a spectral sampling of 0.73 nm. The HS cube is created by shifting the field of view of the camera relative to the scene that is going to be captured, due to the camera being based on the pushbroom scanning technique. In this camera type, the two-dimensional (2D) detector has a dimension of 1004 × 826 pixels, capturing the complete spectral dimensions and one spatial dimension of the scene. For this reason, the camera requires a linear displacement transducer to perform the scanning, achieving a complete HS cube with a maximum size of 1004 × 1787 pixels and 826 spectral bands. Due to the fixed focus of the camera lens, the distance between the lens and the sample must be 40 cm, producing a maximum image size of 129 × 230 mm^2^, where each pixel represents a sample area of 128.7 × 128.7 µm^2^.

**Figure 1:**
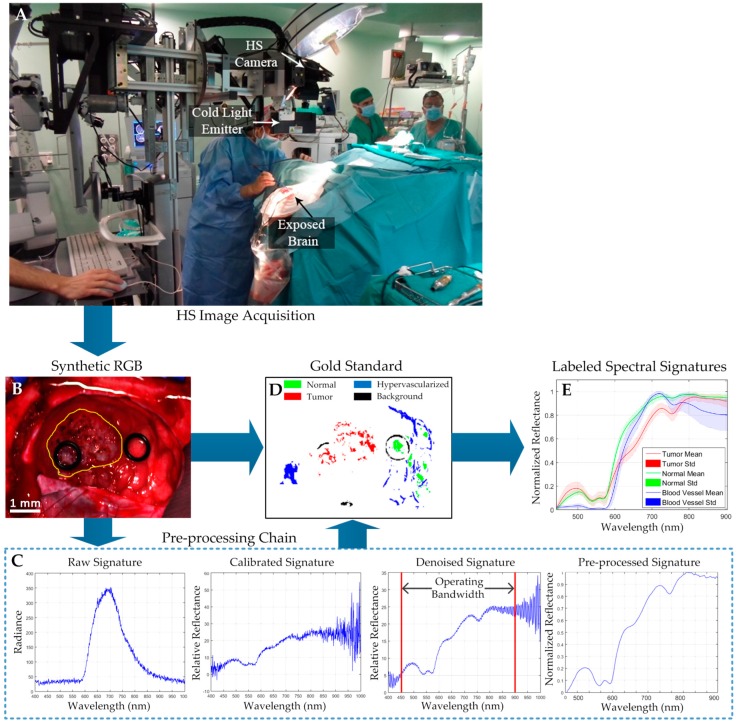
(**A**) Intraoperative hyperspectral (HS) acquisition system capturing an image during a surgical procedure. (**B**) Synthetic red–green–blue (RGB) representation of an HS cube from an *in vivo* brain surface affected by glioblastoma (GBM) tumor (outlined in yellow). (**C**) Input and output spectral signatures of each step of the pre-processing chain employed to pre-process the HS cube. (**D**) Gold standard map obtained with the semi-automatic labeling tool from the HS cube. Normal, tumor, hypervascularized and background classes are represented in green, red, blue, and black color, respectively. White pixels correspond with non-labeled data. (**E**) Average and standard deviation of the spectral signatures of the tumor (red), normal (green), and blood vessel/hypervascularized (blue) labeled pixels.

In addition, an illumination system that is capable of emitting cold light in the range between 400–2200 nm is installed in the acquisition system. A 150-W QTH (quartz-tungsten-halogen) lamp is connected to a cold light emitter via fiberoptic cable. The cold light is required to avoid the high temperatures produced by the QTH lamp in the exposed brain surface. Figure 1A shows the intraoperative HS acquisition system capturing an HS image of the exposed brain surface during a neurosurgical operation at the University Hospital Doctor Negrin of Las Palmas de Gran Canaria (Spain).

### 2.2. In Vivo Human Brain Cancer Database

Twenty-six HS images from 16 adult patients compose the *in vivo* brain HS database employed in this study. Patients underwent craniotomy for resection of intraaxial brain tumor or another type of brain surgery in the routine, clinical practice at the University Hospital Doctor Negrin of Las Palmas de Gran Canaria (Spain). Six of these patients were affected by a confirmed grade IV glioblastoma (GBM) tumor assessed by histopathology. From these patients, a total of eight HS images were acquired while the GBM tumor tissue was exposed. The other 10 patients were registered to obtain normal brain image samples. These patients were affected by other types of primary or secondary tumors or underwent craniotomy for stroke or epilepsy treatment. In the case of the patients with other types of tumors different from GBM, the tumor samples were not included in the study. Written informed consent was obtained from all of the participant subjects, and the study protocol and consent procedures were approved by the *Comité Ético de Investigación Clínica-Comité de Ética en la Investigación* (CEIC/CEI) of the University Hospital Doctor Negrin.

The procedure to acquire the intraoperative *in vivo* brain HS images was accomplished as follows [20]. After craniotomy and resection of the dura, the operating surgeon initially identified the approximate location of the normal brain and tumor (if applicable). Then, rubber ring markers were placed on these locations, and the HS images were captured with markers in situ. Figure 1B shows an example of the synthetic red–green–blue (RGB) representation of an HS image of a GBM tumor where two markers were used to identify the tumor tissue (left marker) and the normal tissue (right marker). The approximate tumor area in the exposed brain is surrounded by a yellow line in the image. After that, tissue samples were resected from the marked areas and sent to pathology for tissue diagnosis. Depending on the location of the tumor, images were acquired at various stages of the operation. In the cases with superficial tumors, some images were obtained immediately after the dura was removed; while in the cases with deep laying tumors, images were obtained during the actual tumor resection.

The raw HS images were pre-processed using the pre-processing chain previously explained in [20]. In summary, this pre-processing chain is based on three main steps: image calibration, image denoising, and image normalization. Figure 1C present the inputs and outputs of each pre-processing step. Next, the goal of each pre-processing step is briefly explained:

(1) **Image calibration**: In this step, the raw data is calibrated using a white reference image and a dark reference image following Equation (1), where $C_{image}$ is the calibrated image, $R_{image}$ is the raw image, and $W_{ref}$ and $D_{ref}$ are the white and dark reference images, respectively. The white reference image is acquired from a Spectralon^®^ tile, which is a type of material that reflects the 99% of the incoming radiation in the full spectral range considered in this work. This white reference is placed at the same location where the patient’s head will be placed during the surgery, thus taking into account all the real light contributions. The dark reference image is obtained by keeping the camera shutter closed, and is used to avoid the dark currents produced by the camera sensor. This calibration ensures the consistency of data and the reproducibility of the algorithm’s results independently to the operating room where the system is used.
  (1) $$C_{image}=100\times \frac{R_{image}-D_{ref}}{W_{ref}-D_{ref}}$$
(2) **Image denoising:** In this step, the high spectral noise generated by the camera sensor was removed, and the dimensionality of the data was reduced without losing the most relevant spectral information. In order to remove the noise in the data, a smooth filter was applied. In addition, the extreme bands, where the noise was particularly high due to the low capabilities of the sensor in these bands, were removed, having an operating bandwidth comprised between 450–900 nm. Finally, the band averaging was performed to reduce the redundant information in the spectral signature, obtaining a final spectral signature composed of 128 spectral bands.
(3) **Image normalization**: In this last step, the spectral signatures were normalized between zero and one to avoid differences in the radiation intensities of each pixel produced by the non-uniform surface of the brain. Different areas of the same brain tissue type could have different radiation intensities, producing misclassifications of the pixels that belong to the same class. In this sense, after performing the data normalization, the classification algorithms will perform a classification based on the shape of the spectral signatures and not based on their amplitudes.

From these pre-processed cubes, a specific set of pixels was labeled using four different classes: tumor tissue, normal tissue, hypervascularized tissue (mainly blood vessels), and background. The background class involves other materials or substances that can be presented in the surgical scenario, but are not relevant for the tumor resection procedure, such as skull bone, dura, skin, or surgical material. This set of labeled pixels was used to train and test the supervised algorithms evaluated in this work. In this application, where human living patients are involved, it is not possible to achieve a complete gold reference map of the captured image with 100% certainty that the pixel represents the established class. To achieve that, a pathologist should analyze the entire brain tissue exposed in the image, and this is obviously not possible due to ethical reasons, since in this case, the neurosurgeon should resect all the tissue exposed in the brain surface (including tumor and normal), causing serious problems to the patient’s health. In other fields such as remote sensing or even in the medical field, but using *ex vivo* or *in vitro* tissue, the complete gold reference generation is easier, but using *in vivo* human samples (and especially from the brain), this task is highly complex and nearly impossible nowadays. Therefore, the operating surgeon labeled the captured images using a semi-automatic tool based on the spectral angle mapper (SAM) algorithm developed to this end [38]. This SAM algorithm is an automated method for comparing the spectra of the pixels of an HS image with a well-known spectrum obtained from a reference pixel [45]. The tool was employed by the corresponding operating surgeon after the completion of the operation to create the gold standard map for each captured HS image. Neurosurgeons were instructed to select only few sets of very reliable pixels instead of a wider set of uncertain pixels. In this tool, the user loads the pre-processed HS cube and selects a reference pixel from the synthetic RGB image. The reference pixel can be at the location where a biopsy was done (where the tumor marker is placed) or at a location far enough from the tumor margins where the surgeon can be quite confident that the tissue is abnormal (in the case of tumor labeling). The tissue inside the rubber ring markers was sent to pathology for a precise diagnosis of the tumor. In the case of normal tissue, hypervascularized tissue, and background classes, the labeling is performed by selecting a reference pixel by the naked eye based on the surgeon’s knowledge and experience. Then, the most similar pixels to the selected reference pixel are highlighted, which is computed by using the SAM measurement, and the user configures the threshold that varies the tolerances on the selected pixels. Once the user concludes that only the pixels belonging to one class have been highlighted, the selected pixels are assigned to that class. Neurosurgeons were instructed to select only a few sets of very reliable pixels instead of a wider set of uncertain pixels.

Figure 1D shows the gold standard map obtained from the HS cube shown in Figure 1B, where the green, red, blue, and black pixels represents the normal, tumor, hypervascularized, and background labeled samples, respectively. The pixels that were not labeled are represented in white. In addition, the average and standard deviation of the labeled pixels from the tumor, normal, and hypervascularized classes are presented in Figure 1E. As seen in Figure 1E, the spectral signature of the hypervascularized class has a large variance in the longer wavelengths. This is because the labeled hypervascularized tissues include both blood vessels and extravasated blood, which can have varied spectral signatures. For example, in Figure 1B,D, the blood vessels are located in the right part of the brain surface, and the extravasated blood can be found in the left part of the HS image. Finally, Table 1 details the total number of labeled samples per class employed in this study and obtained by using the semi-automatic labeling tool based on the SAM algorithm (see Table S1 for more detailed information). As stated before, the operating surgeon performs the labeling by selecting some reference pixels with high confidence of belonging to a certain class. More than 300 pixels were selected as reference for generating the labeled dataset using the labeling tool. For the normal class, all the HS images in the database (26), which belonged to 16 patients, were labeled. In the case of the GBM tumor class, only eight images from six patients were available to label the tumor samples. For the hypervascularized and background classes, 25 and 24 images from 16 and 15 patients, respectively, were labeled (the other images were not suitable for labeling these classes). This labeled data were employed to train the classification algorithms and obtain the quantitative evaluation results following a leave-one-patient-out cross-validation, where the labeled data of the patient to be tested are not used for training the algorithms. The validation of the algorithms and the HS labeled test dataset will be detailed later in Section 2.6. The information of the entire HS cube was employed to qualitatively evaluate the algorithm results in the full brain image based on a visual evaluation of the operating surgeons.

### 2.3. Deep Learning Techniques

A 2D convolutional neural network (2D-CNN) classifier, which was selected because of its ability to incorporate both spectral and spatial components for machine learning, was implemented in a batch-based training approach using the TensorFlow open-source software library [46] on a Titan-XP NVIDIA GPU. From each pixel of interest (each labeled pixel from the gold reference map), an 11 × 11 pixel mini-patch was constructed centered on the pixel of interest. The 2D-CNN was trained with a batch size of 12 patches, which were augmented to 96 patches during training by applying rotations and vertical mirroring to produce 800% augmentation. The 2D-CNN architecture was based approximately on the AlexNet architecture [47]. This is a basic 2D-CNN architecture that was intentionally selected to test the ability of a standard CNN to solve the problem studied in this work. The details of the architecture are presented in Table 2. It consisted of three convolutional layers, one average pooling layer, and one fully connected layer. Gradient optimization was applied to the AdaDelta optimizer with a learning rate of 1.0 and with 200 and 50 epochs for the training data in the binary and multiclass classification, respectively.

In addition, a deep neural network (DNN) was implemented in TensorFlow on a NVIDIA Quadro K2200 GPU and was trained using only the spectral characteristics of the HS samples. This one-dimensional deep neural network (1D-DNN) was composed of two hidden layers with 28 and 40 nodes, respectively, using the rectified linear unit as an activation function. The learning rate was established as 0.1, and the network was trained for 45 and 40 epochs for the training data in the binary and multiclass classification, respectively.

Cross-validation was performed in both algorithms using the leave-one-patient-out method, and the stop criteria for each training epoch number was based on the stabilization of the accuracy to a maximum in the validation group. All of the parameters were maintained for each patient iteration. Furthermore, the training dataset was randomly balanced to the class with the minimum number of samples (the tumor class in this case).

### 2.4. Proposed Deep Learning Framework

**Figure 2:**
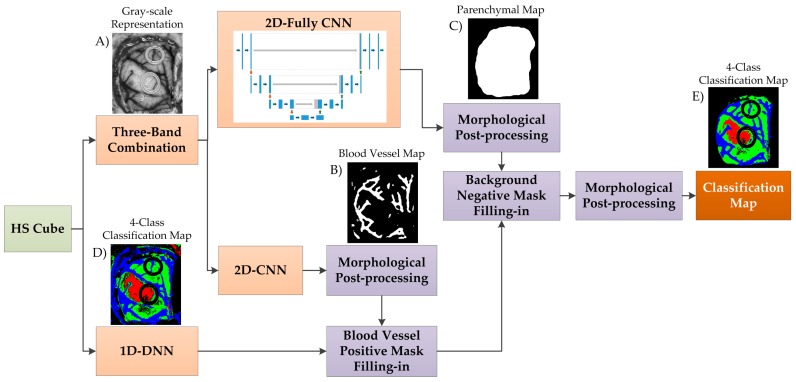
Block diagram of the proposed deep learning framework. (**A**) Gray-scale representation employed as input for the deep learning parenchymal and blood vessel detection algorithms. (**B**) Blood vessel binary classification map. (**C**) Parenchymal binary classification map. (**D**) Four-class classification map obtained from the 1D-DNN algorithm. (**E**) Final four-class classification map generated by the proposed deep learning framework.

The previously described deep learning methods were combined following the framework shown in Figure 2. This framework was developed with the goal of achieving high accuracy results with a reduced execution time. The proposed framework is formed by four main steps: blood vessel detection, parenchymal detection, image classification, and morphological post-processing.

**Figure 3:**
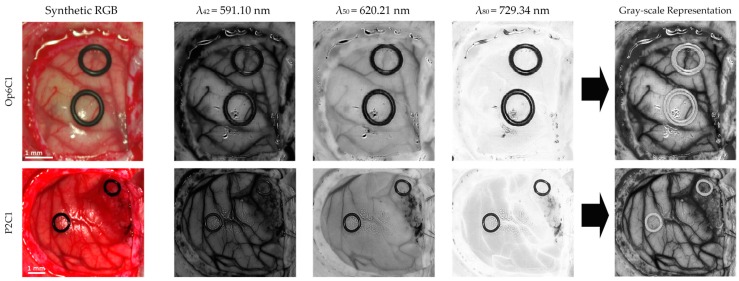
Gray-scale representation image examples and the correspondent three selected spectral channels employed in the three-band combination for the parenchymal and blood vessel detection. A synthetic RGB image is also included for comparison.

Firstly, three spectral channels are selected from the HS cube ($\lambda_{42}=591.10\;\mathrm{nm}$, $\lambda_{50}=620.21\;\mathrm{nm}$, and $\lambda_{80}=729.34\;\mathrm{nm}$), where the subscript of each wavelength indicates the number of the spectral channel in the spectral signature. These spectral channels are linearly combined following Equation (2) to obtain a gray-scale representation (I) of the HS cube where the blood vessels are highlighted (Figure 3). The selection of the most appropriate spectral channels and coefficients was performed empirically, evaluating the contrast of the image by visual inspection. The spectral channel $\lambda_{42}$ was selected because it presents one of the absorption peaks of hemoglobin in the HS image dataset employed in the experiments. Previous works have shown that the hemoglobin concentration absorption peak is normally found between 500–590 nm [48,49,50]. As it can be seen in Figure 3, $\lambda_{42}$ shows a high contrast between the brain tissue and the blood vessels, but the background (especially rubber ring markers) and the specular glare are indistinguishable from the blood vessels. In order to solve this, the spectral channel $\lambda_{50}$ is included in the equation, since it provides a higher contrast between the blood vessels and background. In addition, a reflectance peak of the hypervascularized tissue was found in the spectral channel $\lambda_{80},$ where differences between the brain tissue, background, and the blood vessels are highlighted. As previously mentioned, these spectral channels were linearly combined, obtaining the gray-scale representation image used for the blood vessel and parenchymal area detection (Figure 2A).

(2) $$I=0.7\times \lambda_{42}+0.3\times \lambda_{50}+0.8\times \left(1-\lambda_{80}\right)$$

From this image, image patches of 41 × 41 pixels were generated to be centered on the pixel of interest, and were classified using the 2D-CNN structure previously presented to distinguish between two classes: blood vessels and background. A classification map (Figure 2B) is obtained and optimized using a morphological close operation followed by a morphological open operation with disk structural element of one pixel in radius [51]. Similar results were obtained when using multi-band representations for this classification problem. However, the gray-scale representation was employed in this framework with the goal of achieving real-time processing.

For identification of the parenchymal area, which corresponds to the primary surgical area of the exposed brain, the gray-scale representation was used as input of a 2D fully convolutional CNN. The fully convolutional algorithm implemented was based on the U-Net architecture [52], which was trained for 34 epochs of training data on a manual segmentation of 20 images, which were augmented by a factor of eight with rotations and reflections. The parenchymal map is obtained after applying a morphological close operation followed by a morphological open operation, with a disk structural element of 35 pixels in radius, and a hole-filling operation (Figure 2C). The final model was used to generate the parenchymal maps of eight testing images, achieving a Dice similarity coefficient of 86.5% compared to a manual segmentation generated by the operating surgeon.

In the third step, the HS cube is classified by the 1D-DNN obtaining a four-class classification map (Figure 2D) where the preliminary classification of the normal tissue, tumor tissue, blood vessels/hypervascularized tissue, and background is performed. Then, the blood vessel map is merged to the 1D-DNN classification map through a positive mask filling in, and this result is merged with the parenchymal map using a negative mask filling in.

Finally, in the last step, a morphological open operation, with a disk structural element of one pixel in the radius, is performed to generate the final classification map (Figure 2E).

### 2.5. Traditional Supervised Classification Techniques

**Figure 4:**
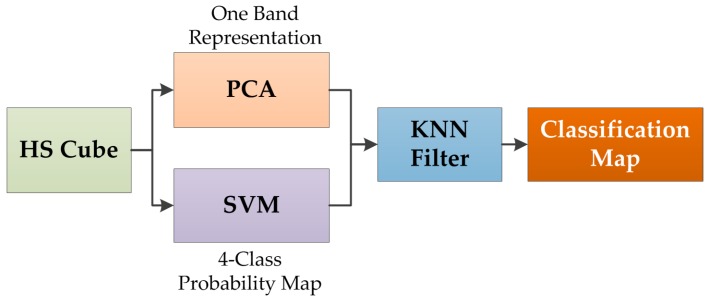
Block diagram of the spatial–spectral supervised algorithm pipeline.

The results of the deep learning algorithms were compared with the results obtained by a spatial–spectral classification algorithm. This supervised algorithm has been already employed for the classification of HSI medical images [38], and it is only employed as a state-of-the-art comparison in this study. In this spatial–spectral algorithm, the HS cube is dimensionally reduced using a principal component analysis (PCA) algorithm to obtain a one-band representation of the HS cube. This one-band representation is used as a guide image to perform a spatial homogenization of the four-class probability map obtained by a support vector machine (SVM) classifier. The spatial homogenization is performed by a K-nearest neighbors (KNN) filter that improves the classification results obtained by the SVM algorithm [38,53]. Figure 4 shows the pipeline of the spatial-spectral supervised classification algorithm. The outcome of this algorithm is a classification map that takes into account both the spatial and spectral features of the HS images.

In addition, different configurations of the SVM classifier were tested using a binary dataset (tumor versus normal tissue) to compare the performance of the algorithms. Linear and radial basis function (RBF) kernels, with the default and optimized hyperparameters, were studied in the binary classification. An exhaustive analysis to find the optimal hyperparameters for both kernels was accomplished, performing a parameter sweep selecting the value that achieved maximum accuracy. Both kernels have a common parameter called *cost* (C). This parameter is the constant of constraint violation that observes whether a data sample is classified on the wrong side of the decision limit. The optimal cost value for the both kernels was $C=2^{6}.$ In addition, the RBF kernel has another specific hyperparameter that is the width of the Gaussian radial basis function, which can be adjusted by the parameter gamma (*γ*). The optimal pair of values (cost and gamma) for RBF was obtained using a grid search method, achieving the maximum accuracy with $C=2^{6}$ and $\gamma =2^{1}.$ The LIBSVM package developed by Chang et al. [54] was employed for the SVM implementation in the MATLAB^®^ R2007a (The MathWorks, Inc., Natick, MA, USA) environment.

### 2.6. Validation

The validation of the proposed algorithm was performed using interpatient classification, i.e., training on a group of patient samples that includes all the patients except the samples of the patient to be tested. This validation methodology is called leave-one-patient-out cross-validation, and consists of extract the data of the patient that is going to be tested from the training dataset, and then repeating this procedure for each patient in the test database. The overall accuracy, sensitivity, and specificity metrics were calculated to measure the performance of the different approaches. Accuracy is defined by Equation (3), where TP is true positive, TN is true negative, P is positive, and N is negative. Sensitivity and specificity are defined in Equations (4) and (5), respectively, where FN is false negative, and FP is false positive. In addition, the receiver operating characteristic (ROC) curve was used to obtain the optimal operating point, where the classification offers the best performance for each patient image, and provide the area under the curve (AUC) metric in the results.

(3) $$Accuracy=\frac{TP+TN}{P+N}$$

(4) $$Sensitivity=\frac{TP}{TP+FN}$$

(5) $$Specificity=\frac{TN}{TN+FP}$$

To compute all of the performance metrics, a bootstrapping method was employed to produce the evaluation metrics with class-balancing and a confidence range. In this method, the class with the lowest number of samples in the test HS image is identified. Next, this number of samples is randomly selected with replacement from all of the remaining classes, and the performance metrics are computed. This procedure is repeated 1000 times, reporting the average value and the 2.5 and 97.5 percentiles to produce the 95% confidence interval. This method was used for each iteration of the leave-one-patient-out cross-validation for the binary mode (tumor and normal samples) and multiclass mode (four classes available in the HSI dataset). The class balancing was necessary to be performed so that all the classes contribute equally to the final metric (AUC, accuracy, sensitivity, or specificity), in order to remove bias from the experiment since the classes were not originally balanced. Table 3 shows the summary of the labeled testing data that was employed for the quantitative and qualitative evaluation of the algorithms. This labeled testing dataset is a subset of labeled data from eight images that composed the complete labeled dataset presented in Section 2.2 and Table 1. In addition, using the classification models generated for each test patient, the classification of the entire HS image was performed to evaluate the results qualitatively. In this study, the classification and visualization systems were evaluated in eight HS images obtained from six GBM patients. The gold standard map of each image was composed by labeled pixels of the four classes (normal, tumor, hypervascularized, and background), allowing the correct computation of the evaluation metrics for each test image performing a leave-one-out cross-validation. The training and testing data were the same in each cross-validation iteration for the deep learning and SVM-based algorithms.

### 2.7. Surgical Aid Visualization System

To evaluate the results obtained by the previously presented supervised classification framework, a surgical aid visualization system was developed using the MATLAB^®^ GUIDE program. In this software, the classification map obtained by the 1D-DNN can be optimized by adjusting the threshold (operating point) where each pixel is assigned to a certain class depending on the probability values obtained for each class. Three threshold sliders were used in the visualization system, which offer the possibility to adjust and overlap the DNN classification results for the tumor, normal, and hypervascularized classes, following the same priority order to overlap the layers.

**Figure 5:**
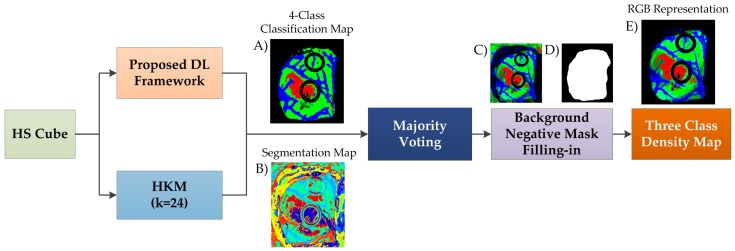
Block diagram of the proposed surgical aid visualization algorithm to generate the three-class density map. A hierarchical K-means (HKM) algorithm and the proposed deep learning (DL) framework were used to generate the maps for the majority voting algorithm. (**A**) Four-class classification map generated by the proposed deep learning framework. (**B**) Unsupervised segmentation map generated by the Hierarchical K-Means algorithm. (**C**) Density map obtained by the Majority Voting algorithm. (**D**) Parenchymal binary classification map obtained in an internal step of the proposed DL framework. (**E**) Three-class density map generated by the proposed surgical aid visualization algorithm.

In this surgical aid visualization system, a processing pipeline based on the proposed DL framework was implemented (Figure 5). This pipeline is able to generate a density map where the three classes (normal, tumor, and hypervascularized tissue) are represented in gradient colors using the classification map of the DL pipeline and an unsupervised segmentation map generated by a clustering algorithm. Concretely, the HS cube is processed by the DL pipeline and a hierarchical K-means (HKM) algorithm, which generates a four-class classification map (Figure 5A) and an unsupervised segmentation map of 24 clusters (Figure 5B), respectively. Both maps are merged using a majority voting (MV) algorithm, i.e., all of the pixels of each cluster on the segmentation map are assigned to the most frequent class in the same region of the classification map [38]. At this point, a new classification map is obtained where the classes are determined by the DL pipeline, and the boundaries of the class regions are determined by the HKM map. In addition, a three-class probability cube is formed by using the probability values of each class in each cluster, where the first, second, and third layers represent the probabilities for the tumor, normal, and hypervascularized classes, respectively. The background class is disregarded when performing the gradient representation of the colors, since it will always be represented by a black color.

Finally, the three-class probability cube is used to generate the RGB density map where each pixel color value (red, green, and blue) is proportionally degraded using the probability values of each layer (Figure 5C). The parenchymal map (Figure 5D) obtained in the DL pipeline is also used at this point to exclusively identify the classification results obtained in the parenchymal area through a negative mask filling-in method, obtaining the final three-class density map (Figure 5E). The algorithm for generating the three-class density map was previously reported [38]. However, this paper uses the DL architecture instead of the supervised spatial–spectral classifier (PCA, SVM, and KNN filtering pipeline), as well as the addition of the parenchymal detection.

## 3. Experimental Results and Discussion

**Figure 6:**
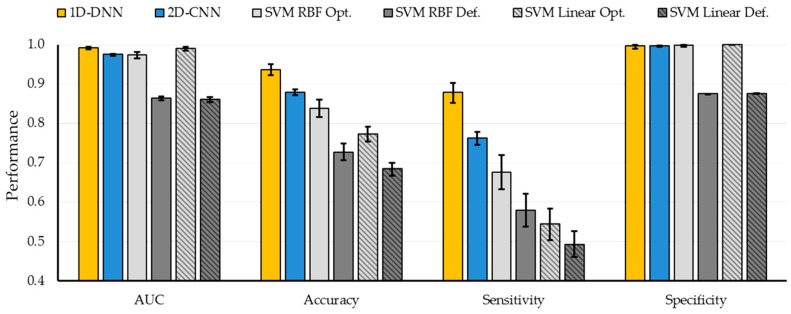
Average results of the leave-one-out cross-validation of the binary dataset obtained for each classification approach using the class-balancing and bootstrapping method with the 95% confidence interval.

The algorithms were tested using eight HS images from six human patients with GBM tumor following the leave-one-out cross-validation method, where the HS data of the patient to be tested was not employed to train the algorithms. In order to evaluate the deep learning methods against traditional SVM-based machine learning algorithms, a binary classification, where only the tumor and normal samples of the database were employed, was performed. Figure 6 shows the average classification results obtained with the six different classification approaches. The SVM-based approaches with default parameters have been included to highlight the importance of the hyperparameter sweep procedure to improve the results. AUC, overall accuracy, sensitivity, and specificity metrics, as well as their respective 95% confidence interval, were computed using the bootstrapping method (see Table S2). The deep learning methods improve the accuracy and the sensitivity compared to the traditional SVM-based machine learning techniques. Particularly, the 1D-DNN achieved the best results, obtaining 94% accuracy, 88% sensitivity, and an AUC of 0.99. Compared to the best SVM-based method, an improvement of 6% in the accuracy is achieved.

**Figure 7:**
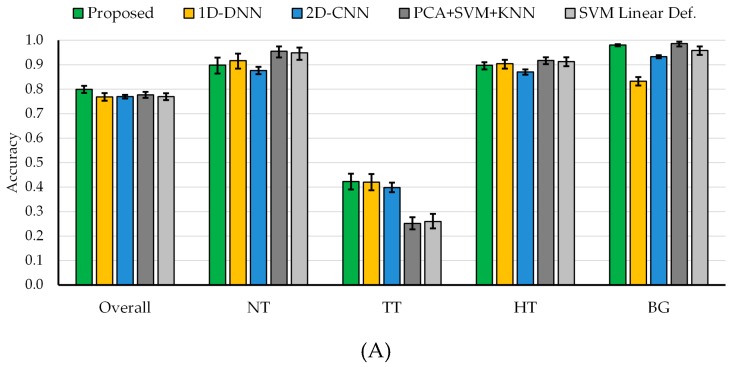

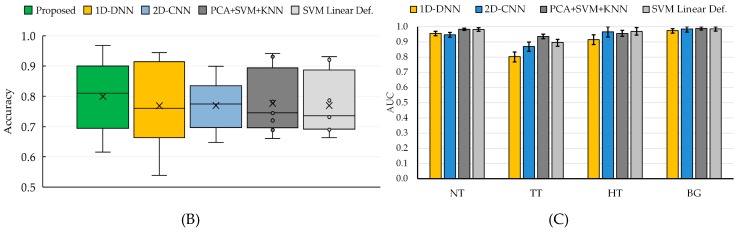
Average results of the leave-one-out cross-validation of the four-class dataset obtained for each classification approach using the class-balancing and bootstrapping method with the 95% confidence interval. (**A**) Overall accuracy and accuracy per class results. (**B**) Boxplot of the overall accuracy results. (**C**) Area under the curve (AUC) results per class. [NT] Normal tissue; [TT] Tumor tissue; [HT] Hypervascularized tissue; [BG] Background.

However, when the four-class dataset is used, the results obtained by both DL techniques are quite similar. Figure 7 and Tables S3 and S4 show the average classification results of the multiclass classification with the 95% confidence interval (shown within the error bar). In this case, the overall accuracy obtained with the 2D-CNN and the 1D-DNN are similar to the traditional SVM-based approaches; however, the sensitivity of the tumor class has been improved by ~16% when using the DL approaches. In this particular case of *in vivo* tissue, it is a challenging task to achieve a high sensitivity in the tumor class.

In order to combine the strengths of both DL techniques, the proposed deep learning framework presented in Section 2.4 was evaluated. Since the main goal of this study is to provide real-time classification during neurosurgical procedures, the development of a fast execution algorithm is critical. As seen in Table S3, the 2D-CNN offers similar results than the 1D-DNN (77% of overall accuracy). However, the required time to transfer and process the HS data by the 2D-CNN (image patches from each pixel with a dimension of 11 × 11 × 128) is significantly greater (~one minute) than the time required by the 1D-DNN (~10 seconds), since the 1D-DNN only exploits the spectral information of the HS cube (pixel-based approach where each pixel has a dimension of 1 × 128). Therefore, in the proposed framework (Figure 2), we use the 1D-DNN as the main classifier, including the 2D-CNN to detect the blood vessels in a gray-scale representation of the HS cube, using image patches from each pixel with a dimension of 41 × 41. Furthermore, another 2D-Fully-CNN is used to detect the parenchymal area of the exposed brain, employing the full gray-scale representation image. These intermediate CNN classification maps take into account the spatial information that is required to homogenize and reduce the false positives in the multiclass classification result obtained by the 1D-DNN (Figure 2).

In this sense, the proposed framework improves the overall accuracy to 80% (3% more than the overall accuracy obtained with the 1D-DNN and the 2D-CNN), although the tumor accuracy remains the same (42%), the detection of the background class is improved to 98% (15% and 5% more than the 1D-DNN and the 2D-CNN, respectively). AUC metrics cannot be obtained for this algorithm, since the optimization process (mixing the blood vessel and parenchymal maps with the 1D-DNN classification map) is performed over the classification map, and the probability map cannot be obtained to compute the AUC. Figure 7A shows the average overall accuracy and accuracy per class results obtained by each algorithm, and Figure 7B presents the boxplot of the overall accuracy results. Although the results obtained are quite similar, it is possible to observe that the proposed framework offers a better generalization in the results, increasing the overall accuracy of the system. Although the tumor sensitivity results obtained in this work need to be further improved, this study shows that DL techniques perform better than the traditional SVM-based algorithms. In this multiclass classification, the SVM algorithm used the linear kernel with default parameters with the goal of comparing the results obtained with a previously published algorithm where this configuration was employed [38]. However, in this case, the multiclass classification was performed, taking into account the interpatient variability following the leave-one-patient-out cross-validation methodology.

For the binary classification scheme, the improvement in the sensitivity performance of the DNN compared to all of the SVM-based algorithms was found to be strongly statistically significant (0.01<p<0.03), using a paired, one-tailed Student’s T-test. This relationship was not found for the CNN compared to all of the SVM-based algorithms, despite the increase in the average performance. Therefore, the method proposed in the paper used the DNN approach. Moreover, the difference in performance between the DNN and CNN was not found to be statistically significant. Additionally, for the multiclass classification scheme, the tumor accuracy performance improvement of the proposed algorithm was found to be marginally statistically significant compared to the DNN alone, PCA + SVM + KNN, and SVM approaches (0.08<p<0.09), using a paired, one-tailed Student’s T-test.

**Figure 8:**
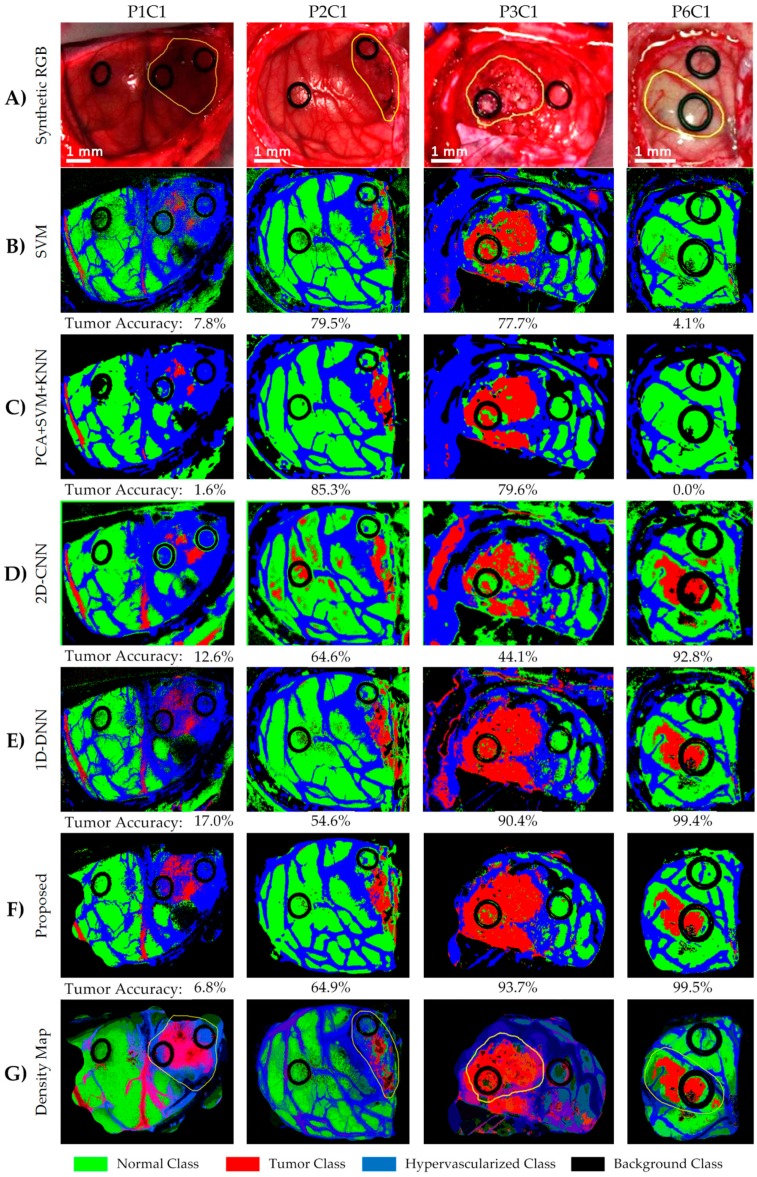
Classification maps of four of the test hyperspectral (HS) images and their respective tumor accuracy below each map. (**A**) Synthetic RGB image with the tumor area surrounded by the yellow lines. (**B**–**F**) Multiclass classification maps obtained with the support vector machine (SVM), principal component analysis (PCA) + SVM + K-nearest neighbors (KNN), 2D convolutional neural network (2D-CNN), one-dimensional 2D deep neural network (1D-DNN), and the proposed framework, respectively. Normal, tumor, and hypervascularized tissue are represented in green, red, and blue colors, respectively, while the background is represented in black. (**G**) Density maps generated using the surgical aid visualization algorithm with the optimal threshold established for the tumor class. In these maps, the colors have been adjusted depending on the probability values obtained after the majority voting algorithm.

Figure 8 shows the classification maps and their respective tumor sensitivity results (below each map) obtained for four test images, which demonstrate that the DL methods significantly improve the results of Patient 6 (P6C1). Furthermore, the results of Patient 1 (P1C1) demonstrate that the proposed DL pipeline (Figure 8F) offers the best results in the detection of the tumor tissue. It is worth noticing that this image was captured under non-optimal illumination conditions, introducing substantial noise in the HS cube. For this reason, the detection of the parenchymal area in this image was not successfully achieved, and the classification results include some false positives (mainly misclassifying blood vessels with the tumor class). These results were qualitatively evaluated by the operating surgeons, who outlined in yellow the approximate tumor area (over the synthetic RGB representation, Figure 8A), taking into account the information provided by the intraoperative MRI and their knowledge and experience in the field. The results obtained with the proposed framework are quite promising, especially in Patient 6, where the location of the tumor was extremely difficult to identify using only the naked eye.

**Figure 9:**
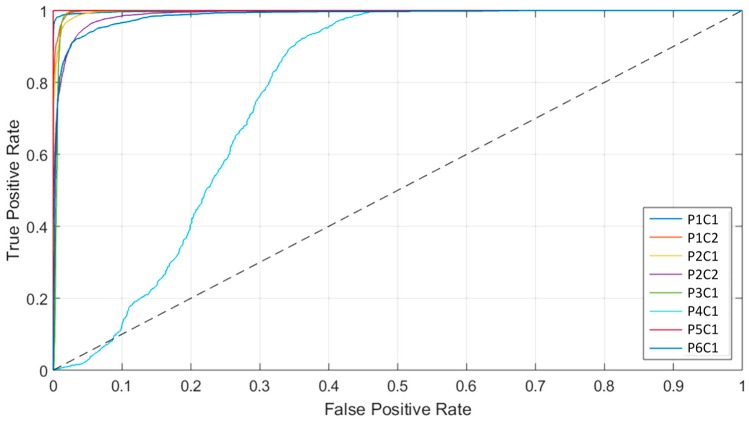
Receiver operating characteristic (ROC) curves of the tumor class for each image of the test dataset generated from the one-dimensional deep neural network (1D-DNN) multiclass results.

Finally, the ROC curves obtained from the basic approaches (Figure 7C and Table S4) show that each class has an optimal operating point where the algorithm is able to classify the samples with high accuracy. These high AUC values indicate that for the majority of cases, there is a classification threshold (operating point) that can achieve accurate classification. However, this optimal threshold has a large variation depending on the image that is classified. As can be seen in Figure S1 and Table S5, the AUC metric is unaffected by the class distribution during the metric computation. The AUC results achieved without the bootstrapping method are within the 95% confidence interval. Furthermore, Figure 9 shows the ROC curves of the tumor class obtained for each image of the validation dataset by using the 1D-DNN, where it is possible to observe that all the images (except P1C1) offer practically optimal ROC curves. In this sense, the development of a surgical aid visualization system is based on the use of the optimal operating point to generate the density maps. Figure 8G shows the density maps of each test image obtained with the proposed surgical aid visualization algorithm (Figure 5), where the optimal operating point was employed to classify each pixel. In these maps, the colors of each class were degraded, depending on the probability values obtained for each class in each cluster. Hence, it is possible to reveal, in some cases, tumor areas that cannot be seen directly in the classification map, as well as remove some false positives produced in the supervised classification.

As it can be seen in these results, the predicted tumor area overlaps well with the gold standard cancer area (yellow contour in Figure 8A). The ability to accurately localize the cancer area can also be seen in the high average AUC values for the tumor class ranging from 0.80 to 0.94 for the algorithms tested in this work. The reason for the low sensitivities is the large optimal threshold differences between the test patients, which is partially due to the lower number of tumor samples in the training set. Additionally, as seen in Figure 1C, the gold standard used for obtaining the quantitative results did not comprise the entire tumor area. Only pixels with a high certainty of class membership were selected, which could have also contributed to the low sensitivity results that do not accurately reflect the efficacy of the proposed method. However, to solve this problem in the proposed surgical aid visualization interface, the operating surgeon can visualize multiple thresholds to determine the sufficient operating point for cancer detection.

**Figure 10:**
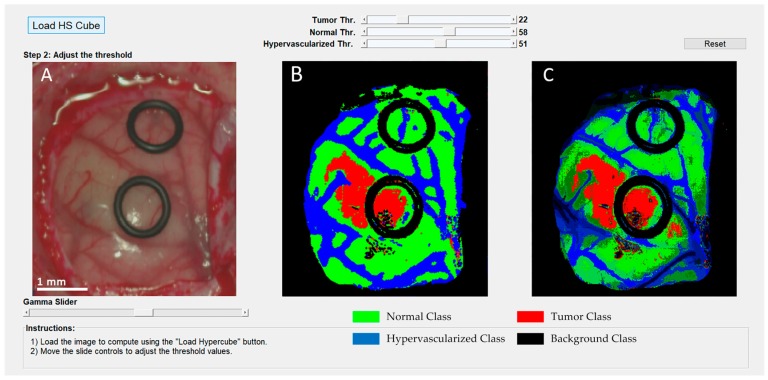
Surgical aid visualization user interface with manual adjustable threshold values. (**A**) Synthetic RGB image generated from the hyperspectral imaging (HSI) cube. (**B**) 1D-DNN classification map generated with the established threshold. (**C**) Density map generated with the new classification map.

Since the automatic computation of the optimal operating point cannot be performed during the surgical procedures due to the absence of a gold standard of the undergoing patient, the surgical aid visualization system was developed based on the manual selection of this operating point. Using the developed user interface (Figure 10), the operating surgeon is able to easily determine the optimal result on the density map (Figure 10C) by manually adjusting the threshold values of the tumor, normal, and hypervascularized classes. These threshold values establish the minimum probability where the pixel must correspond to a certain class in the classification map generated by the 1D-DNN (Figure 10B). After that, the overlapping and the majority-voting algorithms are computed to generate the updated density map. This user interface combines the information provided by the HSI processing and the expertise and knowledge of the operating surgeon.

## 4. Limitations

In this study, the use of HSI to detect high-grade glioblastoma tumors during surgical procedures has been presented. As the main competitor, 5-ALA is a commonly used technique that addresses this problem, providing a margin delineation of high-grade tumors in real-time during neurosurgical procedures. However, 5-ALA has several disadvantages due to the invasiveness of the technique, which produces significant side effects in the patient, and it is not recommended for use in pediatric patients. In contrast, HSI could lead to a potential solution to these problems, being a non-contact and label-free technique that is totally harmless for the patient. In addition, in a previous work [20], it was revealed as a preliminary study that HSI could detect other types of tumors apart from high-grade gliomas, such as a grade I meningioma or a grade II oligodendroglioma. Further experiments should be carried out to confirm these results employing an increased *in vivo* HS brain database with more quantity and types of tumors used to develop and test the classification algorithms. Having enough data, the future of this technology could lead not only to a margin delineation of different types of primary and secondary tumors, but also provide an intraoperative identification of the type and group of the tumor.

The low accuracy obtained in this work for the tumor class indicates that there is an overall error rate of ~58% in the correct identification of the tumor pixels. This result is obtained in multiclass classification, where each class accuracy is defined as the sensitivity obtained for that class. This means that, on average, the proposed method classifies 58% of the tumor pixels as “non-tumor”, i.e., the conjunction of the normal, hypervascularized, and background classes. Taking into account the results obtained in the binary classification where only normal brain and tumor tissues were classified, the sensitivity and specificity results were 88% and 100%, respectively. Therefore, in the multiclass classification, the majority of the misclassifications produced in the tumor class are related to the hypervascularized and background classes. Mainly, the false negatives obtained in the results were tumor pixels assigned to the hypervascularized or background classes. Furthermore, this error is higher or lower, depending on the HS image that is classified. In summary, both the binary and multiclass classification schemes have very high specificity (100% for binary and 90%+ for multiclass), averaging the accuracy of all the non-tumor classes. Therefore, we can conclude that the proposed technique performs well on correctly classifying cases of being disease-free. In other words, the method has a high confidence for “ruling in” cases of disease [55]. In brain cancer resection, an intraoperative guidance system should have a very high specificity to have confidence that the areas resected are not normal brain tissue, which is very valuable for better patient outcomes.

**Figure 11:**
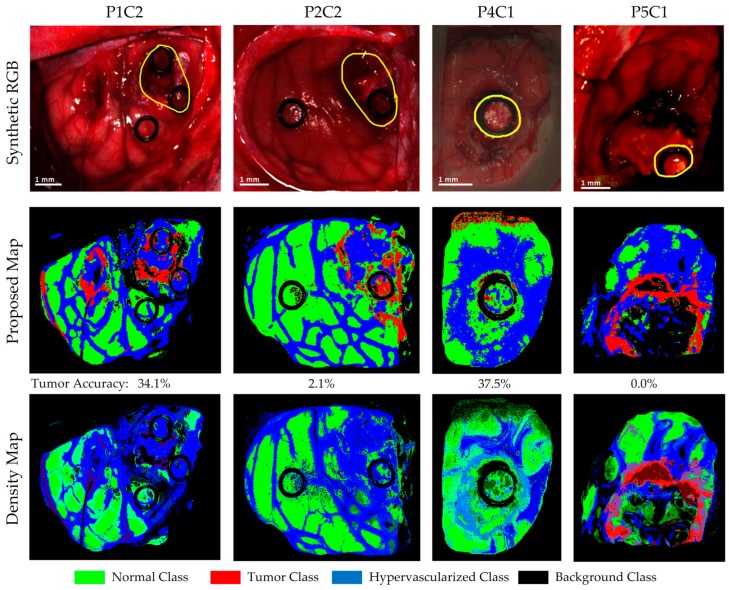
Classification maps of the four test HS images acquired after initial tumor resection and their respective tumor accuracy below each map. In these results, it is possible to observe the limitations of the current system when images are captured during the tumor resection procedure.

**Figure 12:**
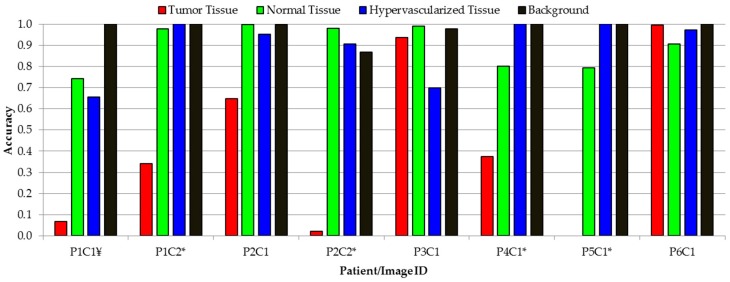
Class accuracy obtained with the proposed method for each test HS image. (*) Indicates the HS images acquired after beginning the resection. (¥) Indicates the image acquired with the tumor exposed in the surface, but under non-optimal illumination conditions.

Four of the HS images employed to compute the evaluation results were acquired after the beginning of the resection. P1C2, P2C2, P4C1, and P5C1 are images captured when part of the tumor had been resected from the superficial tumor (in the cases of P1C2 and P2C2), or when the normal brain of the surface was resected to reveal a deep layer tumor (in the cases of P4C1 and P5C1). The synthetic RGB images and the classification maps achieved for the proposed DL framework as well as the correspondent density map are shown in Figure 11. As it can be seen in this figure, the procedure carried out to perform the resection of the tumor produced several effects in the exposed brain surface (extravasated blood, increase of the vascularization in the surrounded normal tissue, burn marks or presence of surgical serum employed to clean the exposed area) that could produce misclassifications of the tumor pixels. The classification map of P1C2 presents false positives in the left part of the image, where in P1C1, a rubber ring marker was located that was histopathologically diagnosed as normal brain. The effects produced by the resection tools in this area generated the misclassification of the surrounded pixels in this area. Extravasated blood present in the tumor area also generated problems in the classification result of P2C2. Although the surface of the brain was cleaned before the HS image acquisition, the time involved in the acquisition process (~one minute) creates the opportunity for extravasated blood in the image if the tumor area is highly vascularized. This produces misclassifications, especially between the hypervascularized class and the tumor class. Finally, P4C1 and P5C1 present deep-layer tumors, and it was more difficult to get high-quality images due to the limitations of the intraoperative HS acquisition system. Only a few tumor pixels could be accurately labeled with high confidence from these images, as it can be seen in the gold standard reference maps presented in Figure S2. Particularly, the tumor area of P5C1 was captured with a non-optimal focus, which led to the spectral signatures of the tumor area being misclassified with background pixels. In addition, in this classification result, it is possible to observe how the burn marks generated due to the resection tools induces misclassifications between the surrounded normal tissue and the tumor class. In summary, Figure 12 presents the class accuracy results achieved for each HS image in the test dataset using the proposed method. In these results, it is possible to observe that when employing high-quality HS images of the tumor exposed in the brain surface (P2C1, P3C1, and P6C1), the accuracy of the tumor detection is statistically significant. Furthermore, the accuracy of the remaining classes (normal tissue, hypervascularized tissue, and background) is optimal in the majority of the cases. The main problem found here is in the classification of images that were captured under non-optimal conditions or captured after the beginning of the resection, which produces several effects in the tissue that surrounds the resected area. As stated before, the visual analysis of the classification maps obtained with the proposed method (Figure 10 and Figure 11) shows that false positives are clearly obtained in some pixels outside the tumor area (surrounded by the yellow line). P1C1, P1C2, or P5C1 are clear examples. On the other hand, false negatives are also found in the results, where normal tissue or hypervascularized tissue is identified within the tumor area. However, due to the multiform nature of the GBM tumor, these results should be validated through histopathological analysis in further experiments.

In order to solve these problems, further studies should be carried out to evaluate whether the use of the resection tools could affect the spectral signatures of the tissue. In addition, an improvement of the acquisition system, where the HS camera is able to acquire images in real time, is required to obtain high-quality HS images and perform a study that could demonstrate the ability of HSI to provide ongoing feedback during the entire tumor resection process. Finally, in this study, the evaluation of the tumor margin delineation of the system was performed through visual inspection of the classification results by the operating surgeon and taking into account the location of the tumor in the intraoperative neuronavigation system. An exhaustive validation of these classification results should be addressed in future studies in order to confirm the validity of the results obtained. For example, one way to do this could be by performing several biopsies in different points of the tumor area (especially in the margins) that are identified by the system, and carrying out the histopathological analysis of these samples.

Previous experiments have shown that when using SVM-based algorithms, no false positives are presented in the classification results of three normal brain surface images [20]. This is again demonstrated in the binary classification scheme described in this paper, where we obtained a specificity of 100%. Nevertheless, further experiments should be carried out in order to demonstrate that the proposed deep learning approach can obtain similar results when images without brain tumors are classified. This will require the inclusion of more HS images from new patients in the database to achieve reliable results.

It has been demonstrated that the differences in the water content found in the tumor tissue with respect to the normal tissue can achieve a more accurate identification of brain tumors [56]. Raman spectroscopy was employed to study this correlation, demonstrating that in the spectral region comprised between 2817–2985 nm (wavenumber region 3350−3550 cm^−1^), the water content can be quantified and used to discriminate between tumor and normal tissue in oral cancer [57]. Following this approach, the use of HSI should be investigated to demonstrate whether the water content of the tumor tissue could be identified and used to improve the accuracy of tumor identification at lower wavelengths. On the other hand, same investigations should be performed to spectrally analyze the physiological characteristics of hemoglobin in the brain surface. The outcomes of this research could lead into a better identification of the wavelengths that could be used to improve the discrimination of the blood vessels in the brain surface. Furthermore, this hemoglobin characterization could help in the differentiation between the tumor tissue and the surrounded hypervascularized normal tissue, improving the delineation of the tumor margins. 

Finally, in this study, the classification frameworks were evaluated following a pixel-by-pixel approach due to the limitations of the *in vivo* HS brain database. In a pixel-by-pixel approach, two classifiers could be considered statistically indistinguishable if there are two different locations of tumor areas (two tumor sites) in the same image, and both classifiers achieve the same accuracy, but identify pixels in different areas. In this sense, to find the best classification framework, a site-by-site approach should be used to evaluate the results. An aid visualization tool is particularly useful when two or more tumor sites need to be classified, and when the accuracy is different at different tumor sites. Further experiments should be carried out in this direction using a much larger dataset in order to improve the comparison of the classifiers by using a site-by-site approach. While the statistical effects of multiple lesions would likely be significant and warrant further investigation, it is unclear how common this would be in the practice of GBM resection. There are two presentations of multi-lesion GBMs: multi-centric, where several sites exist, and multi-focal, where several foci exist, but belong to one primary site [58,59]. The overall incidence of multiple lesions of GBM range from 0.5% to 20% [58,59,60].

## 5. Conclusions

The work presented in this paper employs deep learning techniques for the detection of *in vivo* brain tumors using intraoperative hyperspectral imaging. Classification methods using 1D-CNN have been demonstrated to have a high accuracy for binary cancer detection in HS images. However, our investigations reveal that both spectral–spatial classification with 2D-CNN and pixel-wise classification with 1D-DNN perform well with no significant difference in accuracy when using a multiclass dataset. We believe that the high spectral resolution of the HS cameras used in this study allows the 1D-DNN to perform with comparable accuracy to CNN methods. Additionally, the limited spatial resolution of the pushbroom cameras (compared with other spectral-scanning HS cameras that provide higher spatial resolution) may also reduce the performance of CNN methods.

In addition, a novel classification framework based on a supervised DL pipeline combined with an unsupervised classification stage has been proposed. This framework was integrated in a user interface with the goal of intraoperatively assisting neurosurgeons during tumor resection, allowing the fine-tuning of the outcome of the algorithm. With the goal of achieving surgical-time results in the operating room and taking into account that both DL methods obtain similar results, the proposed framework uses a DNN for classification, because the CNN requires more execution time (~one minute per HS cube) compared to the DNN (~10 seconds per HS cube).

Moreover, an overall average accuracy of 80% for the proposed method was achieved. Since the training dataset had approximately half of the number of samples in the tumor class compared to other classes, the number of samples for each class was balanced. This reduced the total number of training samples. Additionally, several images were out of focus, not fully illuminated, or presented artifacts due to brain movement during scanning, so the number of high-quality tumor training samples was also limited. In this sense, more data collection with more emphasis on collecting high-quality tumor samples could help balance the dataset and produce better training paradigms for the proposed algorithm, which could potentially lead to better results.

In addition, this data increment in the *in vivo* HS human brain database could allow further experiments where the possibility of employing a reduced pre-processing chain, which only involves the image calibration and normalization, could be evaluated. In this way, it may be possible that more advanced deep learning approaches could learn how to filter out the noise in the spectral signatures, which could lead to an improvement of the sensitivity of the classification results.

The results of this preliminary study show that deep learning outperforms traditional machine learning techniques in the classification of hyperspectral tumor samples. Although further experiments need to be conducted to optimize the deep learning algorithms and compare the multiclass results with the optimized SVM-based classifiers and other algorithms such as Random Forest, PLSDA (partial least squares discriminant analysis), or even linear or non-linear unmixing, the results present a promising starting point for future comparisons.

It is worth noticing that our proposed approach achieves a very high specificity for both binary and multiclass classification schemes, obtaining 100% and ~90%, respectively. These results demonstrate the ability of the proposed approach to achieve high confidence in the correct detection of non-tumor areas, which is ideal in the design of a surgical aid visualization system. Although the binary classification provides better results than the multiclass classification, the use of a four-class classification scheme is required to provide surgeons with an easily interpretable classification map, where different structures are shown, providing more information about the tissue condition thanks to the hypervascularized class. Furthermore, the usage of additional classes has been demonstrated to reduce the misclassifications between hypervascularized and tumor classes, which can be produced when classifying the entire HS cube [38]. In any case, further experiments should be performed to evaluate the ability of the proposed framework to reduce surgical resection margins, employing much more patient data and a multi-centered trial. Furthermore, by using new HS data, the classifiers generated with the previously obtained database could be tested without having to perform the leave-one-patient-out cross-validation. However, the outcomes achieved in this preliminary work demonstrate the feasibility of using hyperspectral imaging as a promising tool for brain surgical guidance.

## Figures and Tables

**Table 1:** Summary of the HS-labeled dataset employed in this study.

Class	#Labeled Pixels	#Images	#Patients
Normal	102,419	26	16
Tumor (GBM)	11,359	8	6
Hypervascularized	38,566	25	16
Background	106,466	24	15
Total	258,810	26	16

**Table 2:** Schematic of the proposed two-dimensional convolutional neural network (2D-CNN) architecture. The input size is given in each row. The output size is the input size of the next row. All convolutions were performed with sigmoid activation and 40% dropout.

Layer	Kernel size/Remarks	Input Size
Conv.	3 × 3/‘same’	11 × 11 × 128
Conv.	3 × 3/‘same’	11 × 11 × 64
Conv.	3 × 3/‘same’	11 × 11 × 92
Avg. Pool	3 × 3/‘valid’	11 × 11 × 128
Linear	Flatten	9 × 9 × 128
Fully-Conn.	-	1 × 10,368
Linear	Logits	1 × 1000
Softmax	Classifier	1 × 4

**Table 3:** Summary of the test dataset employed for the algorithm validation.

Patient ID	Image ID	#Labeled Pixels			
		NT	TT	HT	BG
1	1	2295	1221	1331	630
	2	2187	138	1000	7444
2	1	4516	855	8697	1685
	2	6553	3139	6041	8731
3	1	1251	2046	4089	696
4	1	1178	96	1064	956
5	1	1328	179	68	3069
6	1	1842	3655	1513	2625
Total	8	21,150	11,329	23,803	25,836

^¥^ (NT) Normal tissue; (TT) Tumor tissue; (HT) Hypervascularized tissue; (BG) Background.

## Supplementary Materials

The following are available online at https://www.mdpi.com/1424-8220/19/4/920/s1, Table S1. Detail of the total number of pixels of each class per patient and image of the HS labeled dataset. Table S2. Average results of the leave-one-out cross-validation of the binary dataset obtained for each classification approach using the bootstrapping method with the 95% confidence interval; Table S3. Average accuracy results of the leave-one-out cross-validation of the four-class dataset obtained for each classification approach using the bootstrapping method with the 95% confidence interval; Table S4. Average AUC results of the leave-one-out cross-validation of the four-class dataset obtained for each classification approach using the bootstrapping method with the 95% confidence interval; Table S5. Average AUC results of the leave-one-out cross-validation of the four-class dataset obtained for each classification approach with and without the bootstrapping method. Figure S1. Average AUC results of the leave-one-out cross-validation of the four-class dataset obtained for each classification approach with and without the bootstrapping method. Graphical comparison; Figure S2. Synthetic RGB image, gold reference map, and classification results obtained for each test image using the proposed deep learning framework.
