# Supplementary material for: Deep Learning-Based Framework for In Vivo Identification of Glioblastoma Tumor using Hyperspectral Images of Human Brain
Source: Sensors (Basel). 2019 Feb 22;19(4):920. doi: 10.3390/s19040920 (PMC6412736; doi:10.3390/s19040920)
Supplement: Supplementary file 1 [file sensors-19-00920-s001.pdf]

Deep Learning based Framework for In-Vivo Identification of Glioblastoma Tumor using Hyperspectral Images of Human Brain

Supplementary Material

Table S1. Detail of the total number of pixels of each class per patient and image of the HS labeled dataset.

Patient ID*	Image ID*	#Labeled Pixels				Diagnosis
		NT	TT	HT	BG	
1	2	5,007	0	965	1,992	Normal Brain
2	1	6,061	0	1,727	20,483	Normal Brain
3	1	7,714	0	1,089	0	Normal Brain
4 (1)	1	2,295	1,221	1,331	630	GBM
	2	2,187	138	1,000	7,444	GBM
5	3	10,626	0	2,332	3,972	Normal Brain
6 (2)	1	4,516	855	8,697	1,685	GBM
	2	6,553	3,139	6,041	8,731	GBM
7	1	1,827	0	129	589	Normal Brain
8	1	0	30	64	1,866	GBM
9 (3)	1	1,251	2,046	4,089	696	GBM
10 (4)	1	3,970	0	246	12,002	Normal Brain
	2	349	0	0	2,767	Normal Brain
	3	603	0	234	1,696	Normal Brain
	4 (1)	1,178	96	1,064	956	GBM
	5	2,643	0	452	5,125	Normal Brain
11 (5)	1	1,328	179	68	3,069	GBM
12	1	13,450	0	488	9,773	Normal Brain
	2	4,813	0	958	5,895	Normal Brain
13	1	6,499	0	1,350	1,933	Normal Brain
14 (6)	1	1,842	3,655	1,513	2,625	GBM
15	1	3,405	0	793	5,330	Normal Brain
	2	2,353	0	555	2,137	Normal Brain
	5	969	0	1,637	1,393	Normal Brain
16	1	2,806	0	1,064	3,677	Normal Brain
	2	8,174	0	680	0	Normal Brain
**Total**	**26**	**102,419**	**11,359**	**38,566**	**106,466**	**258,810**

¥ (NT) Normal tissue; (TT) Tumor tissue; (HT) Hypervascularized tissue; (BG) Background.

*The number in brackets corresponds with the identifier of this patient and image in the test database.

Table S2. Average results of the leave-one-out cross-validation of the binary dataset obtained for each classification approach using the bootstrapping method with the 95% confidence interval.

	Average [95% Confidence Interval]			
	AUC	Accuracy	Sensitivity	Specificity
1D-DNN	0.99 [0.99, 0.99]	0.94 [0.94, 0.94]	0.88 [0.88, 0.88]	1.00 [1.00, 1.00]
2D-CNN	0.97 [0.97, 0.97]	0.88 [0.88, 0.88]	0.76 [0.76, 0.76]	1.00 [1.00, 1.00]
SVM RBF Opt.	0.97 [0.97, 0.97]	0.84 [0.84, 0.84]	0.68 [0.68, 0.68]	1.00 [1.00, 1.00]
SVM RBF Def.	0.86 [0.86, 0.86]	0.73 [0.73, 0.73]	0.58 [0.58, 0.58]	0.88 [0.88, 0.88]
SVM Linear Opt.	0.99 [0.99, 0.99]	0.77 [0.77, 0.77]	0.54 [0.54, 0.54]	1.00 [1.00, 1.00]
SVM Linear Def.	0.86 [0.86, 0.86]	0.68 [0.68, 0.68]	0.49 [0.49, 0.49]	0.88 [0.88, 0.88]

Table S3. Average accuracy results of the leave-one-out cross-validation of the four-class dataset obtained for each classification approach using the bootstrapping method with the 95% confidence interval.

	Average Accuracy [95% Confidence Interval]				
	Overall	Normal	Tumor	Hypervascularized	Background
Proposed	0.80 [0.78, 0.81]	0.90 [0.86, 0.93]	0.42 [0.39, 0.45]	0.90 [0.92, 0.89]	0.98 [0.98, 0.98]
1D-DNN	0.77 [0.75, 0.78]	0.92 [0.88, 0.95]	0.42 [0.39, 0.45]	0.90 [0.92, 0.89]	0.83 [0.82, 0.85]
2D-CNN	0.77 [0.76, 0.78]	0.88 [0.86, 0.89]	0.40 [0.38, 0.42]	0.87 [0.88, 0.86]	0.93 [0.93, 0.94]
PCA+SVM+KNN	0.78 [0.76, 0.79]	0.96 [0.93, 0.97]	0.25 [0.23, 0.28]	0.92 [0.93, 0.90]	0.99 [0.97, 0.99]
SVM Linear Def.	0.77 [0.76, 0.78]	0.95 [0.92, 0.97]	0.26 [0.23, 0.29]	0.91 [0.93, 0.90]	0.96 [0.94, 0.97]

Table S4. Average AUC results of the leave-one-out cross-validation of the four-class dataset obtained for each classification approach using the bootstrapping method with the 95% confidence interval.

	Average AUC [95% Confidence Interval]			
	Normal	Tumor	Hypervascularized	Background
1D-DNN	0.96 [0.95, 0.96]	0.80 [0.78, 0.83]	0.92 [0.91, 0.92]	0.97 [0.97, 0.98]
2D-CNN	0.95 [0.94, 0.95]	0.87 [0.86, 0.88]	0.97 [0.96, 0.97]	0.98 [0.98, 0.99]
PCA+SVM+KNN	0.98 [0.98, 0.99]	0.94 [0.92, 0.95]	0.96 [0.95, 0.96]	0.99 [0.98, 0.99]
SVM Linear Def.	0.98 [0.98, 0.99]	0.90 [0.88, 0.92]	0.97 [0.96, 0.97]	0.99 [0.98, 0.99]

Table S5. Average AUC results of the leave-one-out cross-validation of the four-class dataset obtained for each classification approach with and without the bootstrapping method.

	Average AUC (Without Bootstrapping)				Average AUC (With Bootstrapping)			
	Normal	Tumor	Hyper.	Background	Normal	Tumor	Hyper.	Background
1D-DNN	0.97	0.82	0.95	0.99	0.96	0.80	0.92	0.97
2D-CNN	0.98	0.89	0.97	0.99	0.95	0.87	0.97	0.98
PCA+SVM+KNN	0.99	0.96	0.97	1.00	0.98	0.94	0.96	0.99
SVM Linear Def.	0.99	0.92	0.97	1.00	0.98	0.90	0.97	0.99

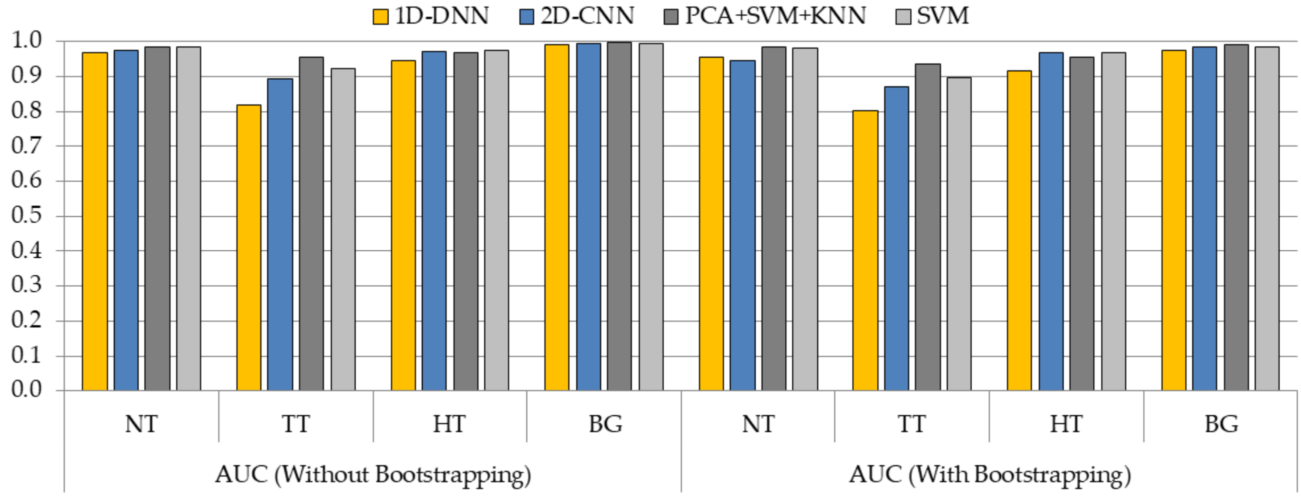

Figure S1. Average AUC results of the leave-one-out cross-validation of the four-class dataset obtained for each classification approach with and without the bootstrapping method. Graphical comparison.

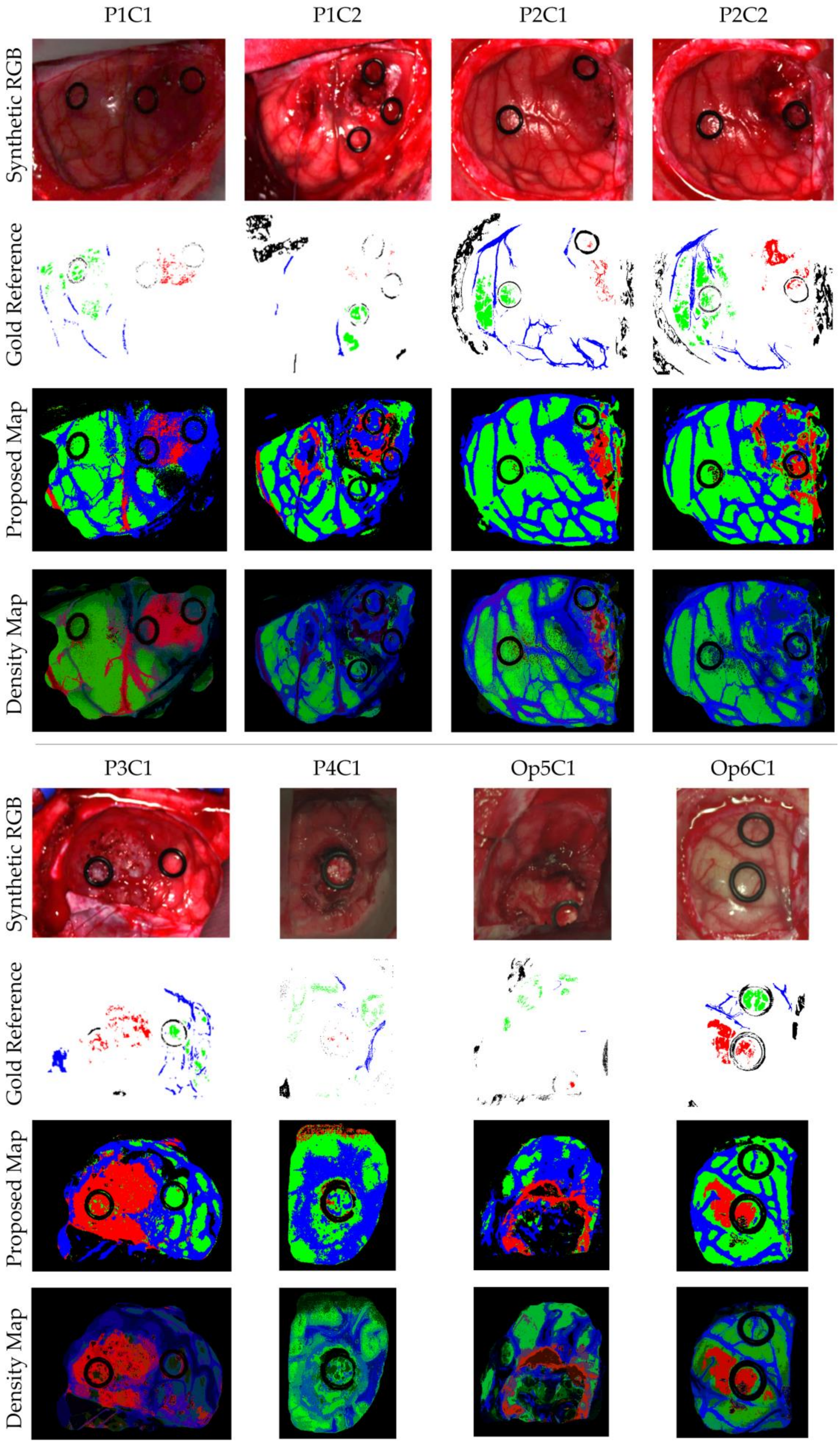

Figure S2. Synthetic RGB image, gold reference map and classification results obtained for each test image using the proposed deep learning framework.
